# Radiation Necrosis in Neuro-Oncology: Diagnostic Complexity and Precision Radiotherapy Strategies

**DOI:** 10.3390/cancers17213542

**Published:** 2025-11-01

**Authors:** Laura Mittelman, James Duehr, Jacob S. Kazmi, Luis O. Vargas, Nora Donahue, John Chen, Sandra Leskinen, Shoaib A. Syed, A. Gabriella Wernicke, Randy S. D’Amico

**Affiliations:** 1 Northwell, New Hyde Park, NY 11040, USA; lmittelman@northwell.edu (L.M.); jduehr@northwell.edu (J.D.); gwernicke@northwell.edu (A.G.W.); 2 Department of Neurosurgery, Lenox Hill Hospital, New York, NY 10075, USA; 3 Donald and Barbara Zucker School of Medicine at Hofstra/Northwell, Hempstead, NY 11549, USA; jkazmi@northwell.edu (J.S.K.); ndonahue@northwell.edu (N.D.); 4 Laboratory for Critical Care Physiology, Feinstein Institutes for Medical Research, Northwell Health, Manhasset, NY 11030, USA; 5 Department of Neurosurgery, Jacobs School of Medicine and Biomedical Sciences, University at Buffalo, Buffalo, NY 14203, USA; lovargas@buffalo.edu; 6 Albany Medical College, Albany, NY 12208, USA; chenj3@amc.edu; 7 College of Medicine, State University of New York Downstate Health Sciences University, Brooklyn, NY 11203, USA; sandra.leskinen@downstate.edu; 8 Department of Radiation Medicine, Lenox Hill Hospital/Northwell, New York, NY 10075, USA

**Keywords:** radiation necrosis, stereotactic radiosurgery, brain metastasis, neuroimaging, workflow, care optimization

## Abstract

**Simple Summary:**

Radiation necrosis (RN) is a delayed complication of cranial radiotherapy and is characterized by progressive tissue injury, inflammation, and vascular compromise. Although advances in stereotactic radiosurgery and fractionated radiotherapy have improved tumor control, RN remains a significant source of morbidity that can mimic tumor recurrence both clinically and on imaging. Relevant mechanisms include endothelial injury, disruption of the blood–brain barrier, and glial-driven edema and necrosis. Diagnosis increasingly relies on advanced MRI modalities and metabolic imaging to differentiate RN from tumor progression, while treatment options include corticosteroids, chemotherapeutics, laser therapy, and, in select cases, surgical resection. Ongoing research emphasizes molecular and connectomic biomarkers to refine diagnosis and personalize management. Understanding RN as a dynamic spectrum of processes rather than a binary entity is essential for optimizing therapeutic decision making in neuro-oncology.

**Abstract:**

Background: Radiation necrosis (RN) is a delayed and potentially debilitating complication of radiotherapy for central nervous system (CNS) tumors. It presents significant diagnostic and therapeutic challenges due to the variable clinical manifestations and overlap with tumor recurrence. Although advances in radiotherapy have improved tumor control, RN remains incompletely understood and inadequately addressed. This narrative review synthesizes current evidence on RN pathophysiology, risk factors, diagnostic strategies, and management approaches. Methods: A literature search was conducted for English-language literature published between January 1990 and December 2024. Studies were included if they addressed RN incidence, diagnosis, treatment, or novel preventive strategies in CNS tumor populations. Relevant findings were synthesized to produce a narrative review summarizing pathophysiology, diagnostic challenges, and treatment strategies. Results: RN results from radiation-induced neurovascular injury, inflammation, and vessel permeability, with incidence ranging from 3 to 26% depending on tumor type, location, and treatment parameters. Risk is influenced by dose, fractionation, cumulative exposure, re-irradiation, and adjuvant therapies. Advanced modalities such as SRS, HFSRT, brachytherapy, proton therapy, and IORT reduce but do not eliminate RN risk. Diagnosis remains challenging despite advanced MRI and PET techniques, with histopathology as the gold standard. Management includes corticosteroids, bevacizumab, surgery, LITT, and experimental therapies. Connectomics-based planning shows promise in minimizing RN by sparing critical brain networks. Conclusions: RN is a clinically significant and multifactorial complication of CNS radiotherapy. Precision treatment modalities and advanced imaging have improved prevention and detection, but diagnostic uncertainty and recurrence risk persist. Integration of connectomics into treatment planning may offer future promise of a reduction in RN-related morbidity by preserving structural and functional network integrity.

## 1. Introduction

Radiation therapy (RT) is a key pillar of standalone or adjunctive treatment in the management of central nervous system (CNS) neoplasms. It is an integral part of a multifaceted treatment approach and therefore is often combined with surgery and systemic therapies. While effective for local control of primary and metastatic brain lesions, RT carries risks of adverse effects, notably radiation necrosis (RN), a delayed complication marked by tissue necrosis within or near the radiation field. RN presents diagnostic and therapeutic challenges due to its heterogeneous presentation and imaging overlap with tumor recurrence [1].

Multiple factors influence RN development, including radiation dose, fractionation, prior radiation, adjuvant therapies, and tumor biology [2]. Pathophysiological mechanisms likely involve radiation-induced DNA damage, vascular injury, chronic inflammation, and aberrant VEGF expression, leading to edema and progressive necrosis [3]. Despite advances in perfusion MRI, MR spectroscopy, and PET, differentiating RN from tumor recurrence remains challenging [4,5].

Although radiation therapy is widely used in CNS tumor treatment, RN development and management remain poorly understood. This narrative review comprehensively synthesizes risk factors and diagnostic challenges for RN in CNS tumor populations. Although the pathophysiology and treatment differ between various primary brain tumors and metastatic lesions, we chose not to distinguish between them due to the shared challenge of RN across tumor types, overlapping mechanisms, and the value of aggregated data. In addition to examining radiotherapy modalities and the potential of connectomics imaging in guiding RN treatment, we propose a comprehensive management algorithm to improve clinical decision making.

## 2. Materials and Methods

**Figure 1:**
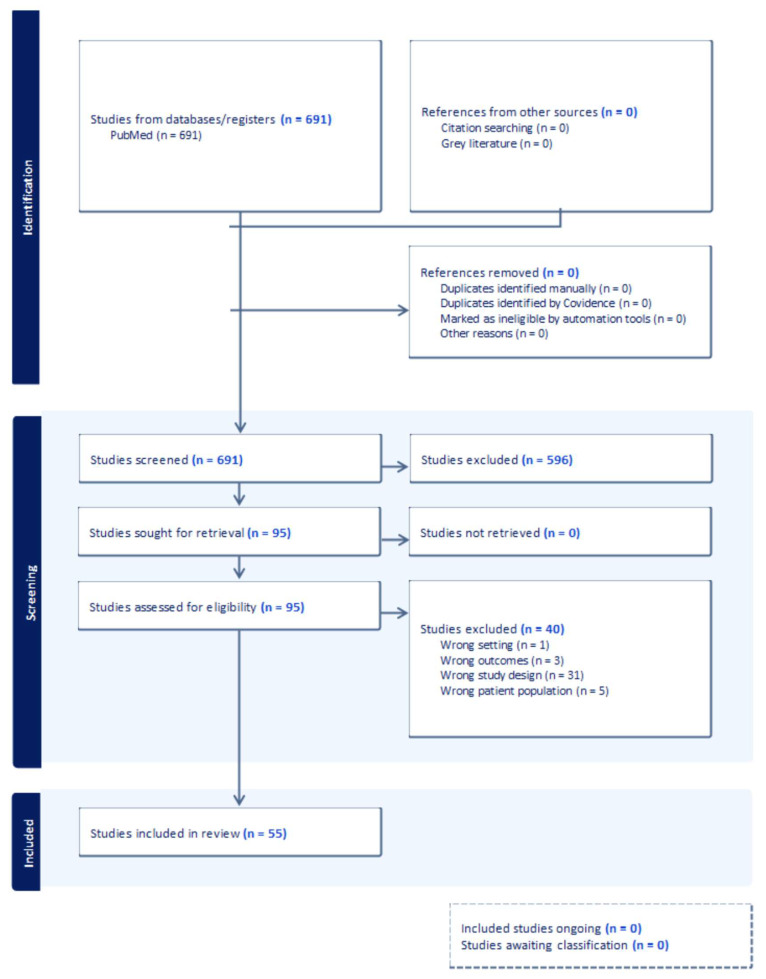
PRISMA-style flow diagram summarizing the study selection process for the radiation necrosis narrative review.

We conducted a literature review on the PubMed database for radiation necrosis (RN) in CNS oncology, covering English-language articles between 1 January 1990, and 31 December 2024, to synthesize the evolving landscape of RN. The following key words and Boolean combinations were used as search criteria: “radiation necrosis” AND (“central nervous system” OR “brain” OR “glioma” OR “brain metastases”) AND (“radiation therapy” OR “stereotactic radiosurgery” OR “proton therapy”) AND (“diagnosis” OR “perfusion MRI” OR “MR spectroscopy” OR “PET” OR “management” OR “treatment” OR “connectomics”). We limited the results to peer-reviewed studies involving CNS neoplasms treated with radiation. Studies were included if they addressed (1) RN incidence or risk factors, (2) diagnostic complexity of RN, (3) therepeutic or management strategies for RN, or (4) emerging radiotherepeutic techniques, including connectomics. Exclusion criteria were: (a) articles limited to non-CNS tumors, (b) studies lacking original data or clinical data synthesis, or (c) articles without relevance to RN in CNS pathology. All eligible studies were narratively synthesized to provide a comprehensive overview of RN pathophysiology, diagnostics, and treatment strategies, with emphasis on connectomics-informed radiotherapy approaches and RN risk minimization. This review followed PRISMA guidelines for reporting, as illustrated in Figure 1.

Populations: We mainly included adult cohorts. Pediatric-only series were excluded from quantitative tables but are discussed narratively when relevant (e.g., proton therapy). We did not conduct a meta-analysis or an age- or sex-stratified synthesis.

## 3. Results

### 3.1. Pathophysiology of RN

Radiation necrosis (RN) results from radiation-induced neurovascular injury, damaging endothelial and glial cells near irradiated tumor tissue. Current theories support that this process leads to fibrinoid necrosis, thrombosis, hemorrhage, and local ischemia [2,6]. Subsequently, hypoxia activates microglia and hypoxia inducible factor-1 alpha (HIF-1α) expression surrounding the necrotic core, upregulating VEGF expression in reactive astrocytes. This drives leaky angiogenesis, vasogenic edema, and an inflammatory cascade that perpetuates tissue damage. Histopathologically, RN features white matter coagulative necrosis, gliosis, capillary collapse, and vessel wall thickening [2,6]. In some severe cases, this progressive inflammatory and ischemic process can extend beyond the initial necrotic focus, leading to progressive demyelination, mass effect, and clinically significant neurological morbidity [7]. RN incidence ranges from 3% to 26% in malignant gliomas or metastatic lesions, and is more closely linked to radiation delivery methods and dosimetric factors (such as total dose, fractionation, and volume) than to tumor pathology itself. However, certain tumor biologies have been identified as possible influency factors. For example, HER2-positive breast cancer and ALK-positive lung adenocarcinoma may increase RN risk, BRAF-mutated melanoma has been linked to a lower incidence [8,9]. Tumor location also matters with lesions near the corpus callosum, periventricular white matter, and infratentorial regions being particularly susceptible, while skull base lesions have lower risk. Differences in capillary permeability, microvascular structure, and treatment protocols likely contribute to these variations [10]. The following sections delve into how various radiotherapy methods impact the development of RN, providing a clear understanding of its molecular drivers.

### 3.2. Risk Factors for RN

RN development is influenced by multiple factors. Clinical risk factors include advanced age, underlying vascular or connective tissue disorders, prior radiation exposure, and adjuvant chemotherapeutic or immunotherapeutic treatments. Radiotherapy-specific factors include the mode of radiation delivery, total irradiated volume, targeted brain region, total dose delivered, fraction size, and the number of fractions. These considerations highlight the importance of individualized radiation planning to optimize therapeutic outcomes while minimizing RN risk. Importantly, both the biologically effective dose and cumulative radiation exposure are directly linked to RN risk in a dose-dependent way. Risk increases even further in patients who undergo re-irradiation or receive multiple radiation treatments—an issue especially important in recurrent CNS disease [8,11,12]. Table 1 summarizes key studies assessing RN risk across different treatment methods, highlighting the relationship between radiation parameters and patient-specific vulnerabilities.

### 3.3. Radiation Therapy Approaches and RN Risk

#### 3.3.1. Whole Brain Radiation Therapy (WBRT)

Whole Brain Radiation Therapy (WBRT), the standard for multifocal brain metastases, increases neurocognitive decline and RN risk due to non-selective exposure [11]. Early clinical trials found WBRT reduced tumor recurrence and mortality due to direct neurological causes but demonstrated no overall survival or functional benefit [21]. Long-term data from the EORTC-NCIC trial (NCT00006353) highlighted chronic radiation toxicity, including potential RN as a significant limitation, despite improved survival [22]. From a radiobiological perspective, this toxicity is driven by the large integral dose and ‘low-dose bath’ to normal tissue. Techniques like hippocampal-avoidance WBRT or partial-brain approaches are linked to better neurocognitive preservation and are expected to reduce RN risk. This is supported by data showing low hippocampal metastasis rates and no compromise in intracranial control [23,24,25,26,27].

Importantly, the Radiation Therapy Oncology Group (RTOG) Phase 1 Dose Escalation Study (RTOG 90-05) established that, for single-dose SRS in previously irradiated tumors, larger tumor size and higher doses markedly increase RN risk, defining the maximum tolerated doses by toxicity as 24 GY (for tumors ≤ 20 mm), 18 Gy (for 21–30 mm), and 15 Gy (for 31–40 mm) [28]. In a study of patients undergoing repeat SRS for recurrent brain metastases, symptomatic RN occurred in 24% of the treated lesions, with the risk strongly linked to the total volume receiving a high dose (V40Gy > 1.6 cm^3^) [29]. In patients with brain metastases who were treated with SRS, prior WBRT was associated with RN development on univariate analysis (HR 2.21, 95% CI 1.28–3.83, *p* = 0.004) [30]. These findings reinforce concerns about long-term toxicity with WBRT [31].

#### 3.3.2. Intensity Modulated Radiation Therapy (IMRT)

IMRT is a high-precision, three-dimensional conformal external beam radiotherapy that delivers targeted radiation to irregularly shaped lesions while sparing adjacent healthy tissue [32]. Despite its advantages, extensive high-dose treatment volumes can still result in RN, particularly in patients with significant tumor burden. As emphasized in the EORTC-NCIC trial, tumor burden and treatment intensity contribute to long-term radiation-induced complications, underscoring the need for optimized dose planning in IMRT to minimize RN while maximizing therapeutic efficacy [22]. Hippocampus avoidance (HA) WBRT, partial brain irradiation, and modern-day brain tumor delivery of radiation by external means predominantly utilize IMRT as the delivery mode. However, unlike SRS, brachytherapy, and hypofractionated stereotactic radiotherapy (HFSRT, also known as FSRS), which all target a narrow margin of healthy brain tissue, IMRT typically involves larger irradiated volumes due to broader treatment margins. Thus, even with superior conformality, IMRT planning for large or complex brain targets should minimize intermediate isodose volumes and integral dose because expanding the normal-brain ‘low-dose bath’ is associated with late toxicities (including neurocognitive decline and RN) [33,34].

### 3.4. Focal Techniques of Radiation Delivery

#### 3.4.1. Stereotactic Radiosurgery (SRS) and Hypofractionated Stereotactic Radiotherapy (HFSRT)

Stereotactic radiosurgery and radiotherapy can be administered using LINAC-based systems (typically with IMRT/VMAT arcs, often utilizing a single isocenter for multiple targets with MLC shaping and frameless immobilization) or on specialized platforms such as Gamma Knife (cobalt-60, with frame or mask immobilization, and multiple isocenters) and CyberKnife (a robotic LINAC with non-isocentric delivery and continuous image-guided tracking). LINAC approaches offer flexibility (intracranial/extracranial), shorter treatment times, and efficient multi-target workflows, although single-isocenter plans may require slightly larger PTV margins to address rotational/setup uncertainties in some scenarios [35]. Conversely, Gamma Knife and CyberKnife typically achieve very high conformity and steep dose gradients—supporting normal-tissue sparing at target edges—albeit with longer treatment durations and, for traditional Gamma Knife workflows, more rigid immobilization requirements [35]. In clinical practice, platform choice depends on lesion number, size, and location, as well as institutional capabilities; importantly, the risk of radiation necrosis most closely relates to dose–volume parameters (e.g., target size and low-to-intermediate isodose volumes such as V10–V16 Gy) rather than the hardware itself, assuming well-optimized planning across systems [35,36,37,38,39,40,41].

The ZAP-X Gyroscopic Radiosurgery system (ZAP Surgical Systems, Inc.) is an innovative, self-shielded, X-ray image-guided platform designed for outpatient stereotactic radiosurgery. Initial clinical data have shown its feasibility, safety, and promising effectiveness in treating intracranial lesions [42]. HFSRT, a related technique, further reduces RN risk by fractionating the radiation over multiple sessions. Published reports show postoperative brain metastases treated with HFSRT have lower RN incidence (7%) compared to single-fraction SRS (23%) [19]. However, these results remain controversial as numerous studies have compared the risk of RN between RT modalities. A 2021 systematic review found no significant difference in RN rates between whole-brain radiation therapy (WBRT), SRS, or combined approaches in post-resection brain metastases (0.93 RR for combined therapy, 95% CI 0.17–5.12%) [43]. In comparison, a recent multicenter cohort study found lower RN rates with HFSRT in postoperative patients with 2–3 cm brain metastases as compared with single fraction SRS (7% versus 23%, respectively, *p* = 0.003) [19]. The radiobiological basis for this reduced toxicity is that fractionation allows late-responding normal brain tissue time to repair sublethal damage between sessions—a capacity that becomes overwhelmed by high-dose single-fraction SRS, especially as the target volume increases [44,45]. An ongoing phase III trial currently underway aims to clarify these outcomes (NCT04114981).

#### 3.4.2. Brachytherapy

Brachytherapy involves implanting radioactive sources directly into or near the tumor, providing precise dose delivery while minimizing broader tissue exposure [46]. Some common forms of brachytherapy include GammaTile^®^ or suture stranded Cesium-131, Iodine-125, Iridium-192-containing TheraSphere™ microspheres, and direct placement of Iridium-192 (^192^Ir) radionuclide seeds [47,48]. This method reduces exposure to surrounding tissue, though RN risk is not completely eliminated. Because continuous low-dose-rate delivery (e.g., I-125, Cs-131) permits ongoing sublethal repair, LDR brachytherapy results in a lower effective dose to normal brain tissue and a reduced risk of radionecrosis compared to high-dose-rate Ir-192 at equivalent EQD2, consistent with classic dose-rate radiobiology and spinal cord/brain tolerance data [49,50].

Early use of Iodine-125 showed RN rates up to 23%, limiting enthusiasm despite improved local control [51]. However, recent advances with Cesium-131 offer lower RN risk due to improved dosimetry and shorter half-life, reducing radiation exposure, especially for larger lesions [52]. Published RN rates range from 1.3% (Cs-131 seeds) to 17.5% (GammaTile). Importantly, brachytherapy with Cs-131 achieves high local control rates in recurrent brain metastases, ranging from 83.3% to 100% at 1-year, with most studies reporting local control rates above 90%, outperforming SRS salvage studies in recurrent brain metastases (60.1–76.6%) [53].

#### 3.4.3. Intra-Operative Radiotherapy (IORT)

Low-energy X-ray intraoperative radiotherapy (LEX-IORT), used as an adjunct to surgical resection in patients with brain metastases, has shown promising results, with one study reporting a 1-year local control rate of 97.1% and a symptomatic radiation necrosis rate of 2.9%. These rates compare favorably to adjuvant stereotactic radiotherapy, where RN rates have been reported as ranging from 8% to over 20% in prior studies [54,55]. Low-energy X-ray IORT delivers the prescribed dose at the cavity surface with an extremely steep gradient—dose drops below 10% within approximately 1–2 cm—so only a small rim of the brain receives a biologically significant dose, which matches clinical reports showing low radionecrosis rates after resection-bed IORT [56,57]. Preliminary data from the prospective single-arm, open-label phase II INTRAMET study (NCT03226483) have reinforced IORT’s safety and efficacy, with a 1-year cumulative local control rate of 94.3% and a mean OS of 37.4 months with no Grade 4 or 5 adverse events. The symptomatic RN rate remained low at 2.9% [54].

#### 3.4.4. Proton and Carbon Ion Therapy

Proton beam therapy (PBT) leverages the Bragg peak phenomenon, enabling highly localized energy deposition at the tumor site with minimal exit dose to surrounding healthy tissue [58]. This precision makes PBT ideal for tumors in eloquent areas especially in pediatric patients [59]. Early studies indicate that PBT may lower RN incidence compared to photon-based therapies, especially in cases requiring high-dose radiation or large treatment fields. However, high costs and limited availability hinder widespread use [38]. Radiobiologically, the Bragg peak allows for precise dose delivery with minimal exit and total dose, reducing late-effect risks—yet higher linear energy transfer (LET) and relative biological effectiveness (RBE roughly 1.1–1.7) at the distal edge, combined with range uncertainty, can cause toxicity if critical structures are in this area. This highlights the importance of robust planning and margin optimization [60,61].

Carbon ion therapy (CIT) combines Bragg peak precision with increased biological effectiveness because of its high linear energy transfer (LET), leading to more DNA damage in tumor cells. These properties have prompted research in glioblastoma, where early clinical data shows promising survival outcomes and acceptable toxicity [39]. CIT’s focused dose delivery and potent tumoricidal effects theoretically reduce RN risk while improving tumor control. Despite its potential, CIT is currently available in only a few specialized centers worldwide, and robust clinical data are needed to confirm its efficacy and safety compared to other advanced radiotherapy modalities [62]. Accordingly, carbon-ion planning must consider high LET/RBE (~2–3) and the distal fragmentation tail, using robust optimization and distal OAR constraints to prevent clinically significant RBE-weighted doses beyond the target [61,63].

### 3.5. Comparative Analysis of RN Risk

RN is a variable complication across radiotherapy modalities (Table 2). Retrospective analyses reveal RN rates of approximately 4.9% in malignant gliomas treated with external beam radiation therapy (EBRT), while Gamma Knife SRS in glioblastoma carries a 15–25% RN risk [20]. In brain metastases, single-fraction SRS is associated with higher RN risk (23%) compared to HFSRT (7%). Brachytherapy-associated RN risk appears to be dose rate–dependent, with low-dose rate (LDR) implants (<30 cGy/hr) associated with a 4% RN incidence, while high-dose rate (HDR) implants (>30 cGy/hr) have been linked to a 27% incidence [64].

It is important to note that many of these reported rates stem from retrospective analyses involving heterogeneous patient populations and varied diagnostic criteria for RN, which contribute to the observed variability. Additionally, while biological behavior and systemic treatment strategies differ between various primary brain tumors and metastatic disease, RN manifests through similar mechanisms of vascular injury and inflammation, and is managed with nearly equivalent strategies. Combining these populations also reflects real-world data and enhances the generalizability of our conclusions.

Importantly, Cesium-131 brachytherapy, due to its advantageous radiobiological features and a short half-life (t_1/2_ = 9.4 days), may provide a lower RN risk for larger lesions compared to high-dose SRS regimens [52]. The ongoing “Radiation One and Done” phase 3 RCT is comparing GammaTile and SRS for brain metastases with post-resection diameter >2.5 cm and may help further delineate these differences [NCT04365374]. This variability highlights the importance of personalized treatment strategies that combine dose, fractionation, modality, and patient-specific risk factors to improve results. While understanding risk stratification is essential, timely diagnosis remains just as difficult. The following section describes key imaging techniques used to distinguish RN from tumor recurrence and pseudoprogression.

### 3.6. Imaging Modalities

**Figure 2:**
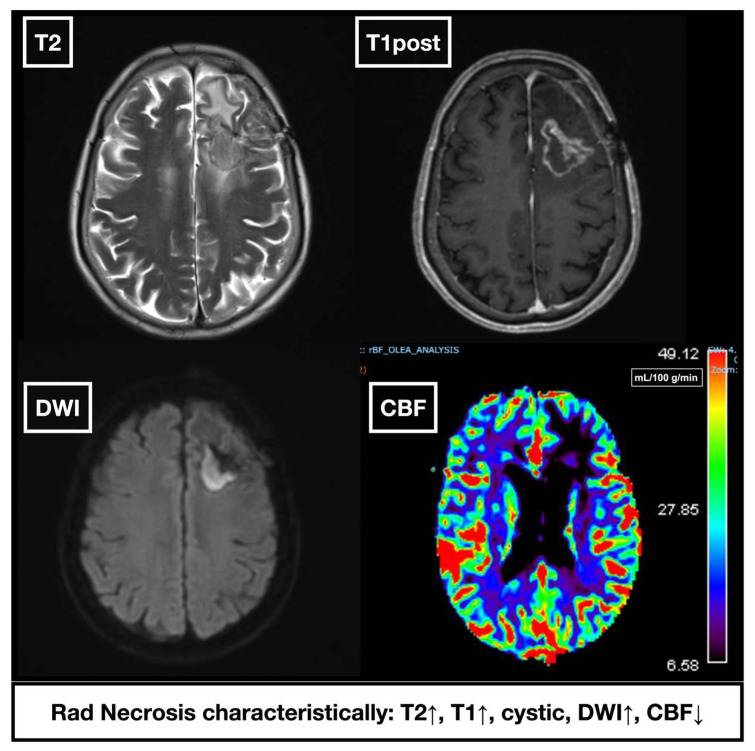
MRI and perfusion imaging features of radiation necrosis. Axial T2, post-contrast T1, and DWI, magnetic resonance sequences, as well as CBF show characteristic findings: T2 and DWI hyperintensity, post-contrast ring enhancement, and decreased cerebral blood flow on perfusion imaging.

Radiographically, RN manifests as an enhancing mass lesion with central necrosis and surrounding vasogenic edema, often located within or adjacent to the irradiated site [65] (Figure 2). On MRI, RN typically displays a “soap bubble” or “Swiss cheese” appearance, representing regions of necrosis intermixed with viable tissue. T2/FLAIR imaging can identify edema, while T1-contrast reveals nodular or curvilinear margins resembling a “spreading wavefront” [66,67].

Advanced imaging techniques, including MR spectroscopy, diffusion-weighted imaging (DWI), and MR perfusion, can aid in distinguishing RN from recurrent tumor, though each has limitations [68]. For instance, DWI demonstrates elevated apparent diffusion coefficient (ADC) values in RN due to necrosis, gliosis, and vessel dilation, in contrast to the dense cellularity of recurrent tumors, with an ADC ratio cutoff of >1.30 exhibiting 86.7% diagnostic accuracy in differentiating RN from tumor progression [69]. Alternatively, PET imagining can be utilized to detect hypometabolic tissue, a hallmark characteristic of RN [67].

Perfusion MRI detects hypoperfusion versus hyperperfusion in recurrent tumors. One method is dynamic susceptibility contrast (DSC) MRI, which measures relative cerebral blood volume (rCBV) and relative cerebral blood flow (rCBF) using normal appearing white matter as a reference region [70]. Alternatively, arterial spin labeling (ASL) provides CBF values in mL/100 g/min without contrast administration [71]. RN can be detected by these imaging modalities through reduced rCBV/rCBF values. MR perfusion techniques can aid in differentiating recurrent tumor from RN utilizing quantitative CBF values of known recurrent tumor tissue, which demonstrates hyperperfusion and neovascularity [72]. Unfortunately, technical limitations compromise the utility of MRI modalities in the diagnostic setting, notably the potential for MR perfusion to be confounded by edema and prior surgery [47,48].

On the other hand, MR spectroscopy provides the choline-to-N-acetylaspartate (Cho/NAA) ratio, which is the most reliable metabolic marker for the discrimination of radiation necrosis and recurrent tumor [73]. Characteristic metabolic changes, notably an elevation in the choline (Cho) to creatine (Cr) ratio and the Cho to N-acetylaspartate (NAA) ratio, and a concomitant decrease in NAA/Cr, reflect the characteristics of malignant tissue, such as proliferation, pathological neuronal membrane integrity, and anaerobic metabolism [74,75]. Contrarily, RN is characterized by persistent lipid signals and decreased choline levels. Elevated lipid signals are also characteristic of pseudoprogression (PsP), while lower lipid signals are present in tumor progression (TP), which in conjunction with metabolite levels can help distinguish between PsP, TP, and RN [75]. 3-dimensional MRS further enhances diagnostic accuracy by mapping metabolic heterogeneity across the entire lesion and localizing focal areas of elevated choline and improving detection of viable tumor within complex post-treatment tissue [76]. Although partial volume effects may diminish contrast in heterogeneous voxels, precise voxel positioning and smaller voxel dimensions can effectively mitigate this limitation. In sum, the largest limitation of MR spectroscopy is its lack of standardization and spatial resolution, and ADC values may overlap between radiation necrosis and pseudoprogression [77].

Postoperative imaging interpretation is particularly challenging. Postsurgical changes such as gliosis, cavity collapse, hemorrhage, or reactive enhancement often mimic or obscure RN. Early imaging may yield false positives due to inflammation, while delayed imaging may miss early RN progression. Advanced techniques like perfusion MRI or spectroscopy can be distorted by surgical artifacts or edema. Consequently, imaging must be interpreted in context, and a lower threshold for repeat imaging or multimodal evaluation, (including PET or radiomics), may be warranted.

### 3.7. Differentiating RN from Pseudoprogression and Tumor Progression

**Figure 3:**
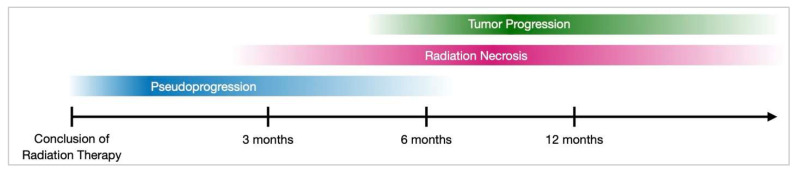
Timeline of outcomes after radiation therapy. Pseudoprogression (blue), represents the earliest stage and shows an increase in tumor size on imaging. Later, the tumor may also exhibit early anti-angiogenic effects, characterized by a temporary reduction in enhancement around the tumor. Radiation necrosis (purple) reflects the necrotic effects of radiation on healthy tissue. Tumor progression (green) typically occurs several months later.

In clinical practice, the main challenge is often distinguishing tumor progression from radiation necrosis and pseudoprogression, as all three can have similar imaging and clinical features after radiation therapy. This diagnostic uncertainty significantly affects treatment decisions, since strategies vary greatly between true progression and treatment-related effects [78]. Pseudoprogression, a transient increase in lesion enhancement following treatment, reflects an exaggerated therapeutic response rather than true disease progression or necrosis and often resolves spontaneously without intervention [79]. Pseudoprogression is most common within the first three months following radiation therapy (Figure 3). In contrast, RN often arises six months to several years post-treatment and may worsen over time [80]. Clinically, pseudoprogression is frequently asymptomatic, whereas RN and tumor progression or recurrence often present with neurological symptoms [81].

Perfusion MRI, using metrics such as cerebral blood volume (CBV), aids clinicians in further differentiation, as CBV values are significantly higher in recurrent gliomas compared to RN (mean relative CBV of 2.38 ± 0.87 vs. 1.57 ± 0.67, respectively). Additionally, lesions with low ferumoxytol-based CBV values (0.7 ± 0.2) are more likely pseudoprogression, while higher values (10.3 ± 3.4) suggest tumor recurrence [66].

### 3.8. Diagnostic Considerations

**Figure 4:**
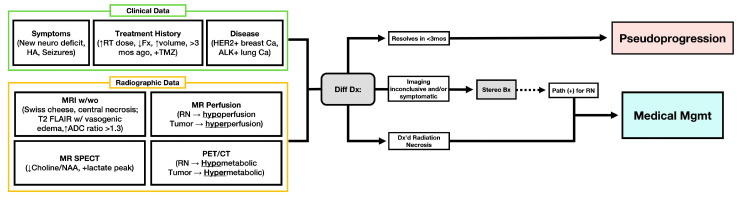
Diagnostic algorithm for differentiating radiation necrosis from tumor progression following radiation therapy for CNS neoplasms in a multidisciplinary clinical setting. ADC, apparent diffusion coefficient; ALK+, anaplastic lymphoma kinase positive; Diff Dx, differential diagnosis; F/u, follow-up; Fx, fractions; HER2+, human epidermal growth factor receptor 2 positive; MR SPECT, magnetic resonance single-photon emission computed tomography; MR w/wo, magnetic resonance imaging with or without contrast; PET/CT, positron emission tomography/computed tomography; PP, pseudoprogression; RN, radiation necrosis; RT, radiation therapy; TMZ, temozolomide; T2 FLAIR, T2 fluid-attenuated inversion recovery; Path (+), pathology positive.

The clinical presentation of RN is variable and ranges from focal neurological deficits to generalized symptoms such as cognitive decline, headache, and seizures [9]. As discussed above, imaging techniques are often unreliable for distinguishing RN from residual tumor, metastatic progression, and pseudoprogression [3]. As a result, the gold standard for diagnosis of RN is histopathological evaluation of biopsy specimens [82]. However, biopsy is an invasive procedure with associated procedural risks, including sampling error, and is infrequently performed for RN. Consequently, a multidisciplinary approach that integrates clinical history, advanced imaging, and patient-specific factors is essential for accurate diagnosis [2,82]. A diagnostic algorithm for distinguishing radiation necrosis from tumor progression in a clinical setting is proposed in Figure 4.

#### 3.8.1. RN in the Context of Immunotherapy

Checkpoint inhibitors, increasingly used in CNS metastases, may amplify RN risk by enhancing inflammatory responses to prior radiation. Retrospective studies suggest increased RN in patients receiving SRS within two weeks of immunotherapy. Pathologically, these cases may show more lymphocytic infiltration and edema. Clinically, they can present with steroid-refractory symptoms. Differentiating RN from immune recall effects or true progression remains a challenge, underscoring the need for prospective studies and immune-sensitive biomarkers.

#### 3.8.2. Biomarkers to Differentiate RN from Recurrence

Novel biomarkers may support imaging in distinguishing RN from recurrence. Circulating neurofilament light chain (NfL) and glial fibrillary acidic protein (GFAP) reflect axonal and astrocytic injury, respectively, and may be elevated in RN, though not specific. Radiomic approaches using MRI-derived texture and shape features have shown >85% accuracy in early studies. These tools, while investigational, may reduce reliance on biopsy and enhance individualized management in the future.

### 3.9. Management Strategies

**Figure 5:**
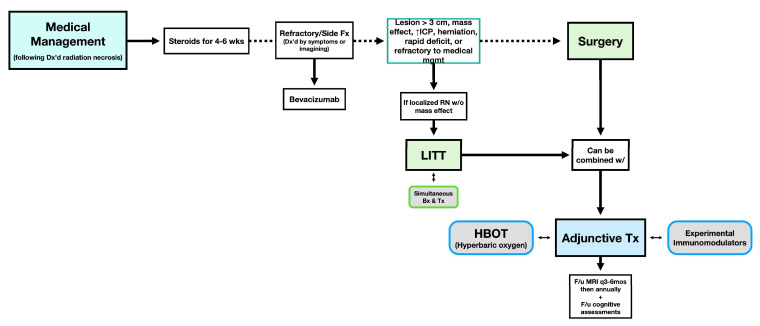
Management workflow for diagnosed radiation necrosis following radiation therapy for CNS neoplasms in a multidisciplinary clinical setting. Adjunctive Tx, adjunctive therapy; HBOT, hyperbaric oxygen therapy; ICP, intracranial pressure; LITT, laser interstitial thermal therapy; RN, radiation necrosis; Tx, treatment; Fx, fractions; F/u, follow-up.

Managing RN requires a tailored, multidisciplinary approach that balances symptomatic relief with long-term outcomes. Figure 5 depicts a streamlined management workflow for diagnosed RN. This framework is supported by the evidence-based strategies detailed below.

#### 3.9.1. Medical Therapies

Corticosteroids: Corticosteroids such as dexamethasone are traditionally first-line treatment due to their ability to reduce local edema and stabilize vascular permeability to reduce blood–brain barrier leakage [6]. However, prolonged use is limited by adverse effects such as hypertension and immunosuppression, which can severely impact an individual’s quality of life, particularly in patients who are undergoing adjuvant chemo- or immunotherapies [6]. A short, tapered course of the lowest dose that provides symptomatic relief is recommended to avoid rebound edema [83]. Retrospective data in patients with brain metastases demonstrate that although steroids provide temporary improvement with a median progression-free survival of 2.9 months, recurrence of RN occurs in nearly 50% of patients after discontinuation [83,84].

Bevacizumab: Bevacizumab, a monoclonal anti-VEGF antibody, targets the VEGF-mediated vascular permeability that underlies RN [6]. Its convenient administration, long half-life, and minimal side effects make it a reliable treatment option [85]. Notably, repeated courses of bevacizumab show high efficacy, with recurrent symptom relief in 90% of patients treated with subsequent cycles [84]. Clinical studies demonstrate bevacizumab not only reduces edema more effectively than corticosteroids (65.5% vs. 31.5% [85] positive response rate; *p* < 0.001), but is also associated with significantly improved outcomes (*p* = 0.003) [86]. A separate retrospective study analyzing clinical data from 45 patients treated for symptomatic CNS RN using bevacizumab (7.5 mg/kg) administered every three weeks for up to four cycles found that while MRI scans showed edema reduction rates of 49–63%, 15% of patients exhibited no radiologic response and 34% experienced lesion recurrence, highlighting transient effects with minimal survival benefit [87]. While generally well-tolerated, Bevacizumab’s primary side effect is hypertension, which is manageable with antihypertensives [88]. Optimizing bevacizumab administration involves using low doses for short-term symptom relief, as excessive or prolonged use may lead to vessel pruning, localized ischemia, and necrosis recurrence [85,86].

Importantly, recent prospective registry data suggest that bevacizumab monotherapy is as effective as combination therapy with corticosteroids, suggesting that the combination provides no additional benefit [89].

#### 3.9.2. Non-Pharmacological Interventions

Surgical Resection: Surgical resection of necrotic tissue is typically recommended for patients with significant mass effect, increased intracranial pressure, refractory neurological symptoms, or progression despite conservative management. Rates of success are high, with over half (54%) of patients reducing or discontinuing steroids postoperatively and up to 83% of patients reporting clinical improvement [90]. Post-operative edema typically resolves within 2–4 weeks, with low recurrence rates [91]. Notably, no prospective trials have evaluated the efficacy of surgical resection for cerebral radiation necrosis [92].

Laser interstitial thermal therapy (LITT): LITT is a minimally invasive alternative that allows for targeted ablation of necrotic brain tissue while enabling concurrent stereotactic biopsy for accurate diagnosis. Early studies report neurological improvements and reduced seizure frequency in 50–70% of patients and improved functional status reported in 70–80% of cases, suggesting that LITT may eventually challenge current first-line medical therapies [93].

#### 3.9.3. Experimental and Adjunct Therapies

Emerging therapies, such as hyperbaric oxygen therapy (HBOT) and immunomodulatory agents, are under investigation. HBOT has shown promise in stabilizing necrotic progression and promoting tissue repair, with small studies reporting improvement in 70–80% of patients. However, these findings must be cautiously balanced against the competing risk of stimulating tumor growth [94]. Pentoxifylline (Trental) and vitamin E have also been noted as additional treatments for RN, with certain studies indicating radiological improvements and decreased RN volume when combined with other therapies [95]. Similarly, novel immunotherapies, including checkpoint inhibitors and novel agents targeting vascular repair pathways, are being explored as adjuncts to conventional treatment [3].

A concise summary of therapeutic options, mechanisms of action, expected duration of response, and limitations is provided in Table 3.

### 3.10. Connectomics in Tailoring Radiation Therapy: Tailoring Treatment to Minimize RN

Conventional RT planning focuses on targeting tumors while sparing gross anatomical structures, but often overlooks the brain’s complex network connectivity [96]. Emerging evidence suggests that radiation exposureto functionally significant networks—beyond classical eloquent areas—may contribute to RN-related cognitive and behavioral deficits [97].

Advanced imaging techniques, such as diffusion tensor imaging (DTI) and blood oxygenation level-dependent (BOLD) signals in conjunction with resting state functional MRI (rsfMRI), enable personalized connectome mapping of the brain’s structural and functional networks. Integrating these maps into RT planning enables precise dosimetry, minimizing radiation exposure to critical connectivity hubs. This approach may better preserve motor, sensory, and language functions while protecting networks involved in cognitive processing. Recent studies have shown that incorporating DTI-based fiber tracking into RT planning is feasible for protecting critical brain structures. For example, Diehl et al. integrated navigated transcranial magnetic stimulation (nTMS)-derived DTI reconstructions of the corticospinal tract into adjuvant RT plans for patients with resected brain metastases [98]. This method significantly reduced the dose to motor tracts—especially in high-dose regions—without compromising target coverage.

In a different study, multi-voxel pattern analysis was used to demonstrate that pre-treatment functional connectomics can predict which breast cancer patients will experience reduced quality of life (QoL) one year later. Functional connectivity in prefrontal regions, including the paracingulate gyrus, superior frontal gyrus, and frontal pole, was significantly linked to long-term QoL outcomes [99]. These results emphasize the potential of connectome-based neuronal biomarkers to identify at-risk patients and guide interventions, such as cognitive behavioral therapy, to maintain QoL. Longitudinal studies are necessary to confirm the link between network-level radiation exposure and clinical outcomes, but early data indicate that protecting high-centrality regions may reduce RN-related complications.

### 3.11. Future Directions

First, connectome analysis offers an opportunity to move beyond lesion-based paradigms toward network-level understanding of radiation-induced injury. Prospective studies incorporating rsfMRI and DTI can delineate alterations in structural and functional connectivity both within and beyond the irradiated region. Quantitative connectome metrics may serve as biomarkers of cognitive decline, diaschisis, and treatment response. Integration of connectomic data into radiation planning could further enable identification and preservation of network “at-risk” regions prior to therapy. Second, clinically meaningful biomarkers are needed to distinguish radiation necrosis from tumor recurrence and to predict treatment response. Longitudinal studies should evaluate plasma and cerebrospinal fluid markers, including circulating tumor DNA, alongside imaging-based radiomic signatures derived from MRI and PET. Cross-validation of molecular and imaging biomarkers within prospective cohorts is essential to establish reproducible diagnostic thresholds and facilitate clinical translation. Lastly, preclinical models and human tissue analyses remain crucial for elucidating microvascular, glial, and immunologic mechanisms underlying necrosis. Linking histopathologic features with imaging and connectomic correlates may uncover mechanistic biomarkers and guide development of targeted neuroprotective strategies. Overall, future efforts should emphasize multicenter registries and standardized imaging protocols, enabling accurate assessment of lesion evolution, treatment response, and long-term cognitive outcomes.

## 4. Limitations

An important limitation of our evidence is the reliance on retrospective studies, which are inherently subject to selection bias and heterogeneous samples. Many analyses include mixed patient populations without consistent stratification by factors such as age (pediatric vs. adult), sex, or primary tumor type, and utilize variable diagnostic criteria for radiation necrosis. In addition, incomplete longitudinal follow-up and differences in imaging modalities or treatment paradigms may lead to over- or underestimations of true incidence and complicate direct comparison across radiotherapy modalities. While our review highlights key patterns, prospectively designed and standardized studies are needed to better delineate risk and refine RN management strategies.

## 5. Conclusions

Radiation necrosis remains a significant challenge in the treatment of CNS tumors. Its development is influenced by a multitude of clinical and radiotherapy-specific factors. Advancements in imaging and treatment modalities, including precision techniques such as SRS, HFSRT, and proton therapy, as well as emerging strategies like connectomics-guided radiotherapy, offer promising avenues for minimizing radiation-related risks. Future research should focus on validating these novel approaches in large, prospective trials to refine treatment protocols and improve patient outcomes. The proposed management algorithm described here serves to streamline a workflow to improve patient outcomes and provide a foundation for the implementation of new therapies.

## Figures and Tables

**Table 1:** Characteristics of included studies for analyzing incidence and risk factors of RN between brain metastases and primary glioma patients.

Author Year Study Type Sample Size	Primary Brain Tumor or Metastasis	Histology (Tissue of Origin for Metastasis)	Type of Radiation Delivery	Radiation Dose	Additional Therapies	Radian Necrosis Incidence	% Radiographic RN Only	% Symptomatic RN	Follow-Up Duration	Time to SRS After Surgery (Days)	Time from SRS to RN	RN Management Strategies	Factors Assessed
Demetz et al., 2023 [10] Retrospective *N* = 205	Primary Brain Tumor	All benign neoplasms (vestibular schwannoma, meningioma, glomus jugulare tumors, ependymoma, schwannoma of other cranial nerves, others)	LINAC based SRS (unspecificed number of fractions)	12–18 Gy (median 13 Gy)	Steroid taper (routine)	15.6% (total)	53%	47%	Mean 42 months, standard deviation 16.3 months, range 0–192 months	N/A	Median 10 months	Dexamethasone in 12 cases, bevacizumab in 2 cases, surgical resection in 2 cases	Tumor location, applied radiation dose
Kerschbaumer et al., 2021 [13] Retrospective *N* = 388	Mixed	Mixed (NSCLC, melanoma, breast, renal, unknown met, vestibular schwannoma, meningiomas, ependymoma, glomus tumors, gliomas)	Single staged LINAC based SRS	14–25 Gy (median 16 Gy)	Steroid taper (routine)	15.7% (total)	53%	47%	Mean 24 months, range 0–192 months)	N/A	Median 8 months (range 1–41 months)	Medical management for 23, surgery for 2, 2 palliative	Tumor diameter, radiation dose
Ruben et al., 2006 [14] Retrospective *N* = 426	Primary Brain Tumor	Glioma	EBRT	16–60 Gy (median 50 Gy)	SRS (12%), Conformal EBRT (0.5%), interstital brachytherapy (0.7%), chemotherapy	4.90%	Did not specify	Did not specify	Did not specify	N/A	Mean 11.6 months, range 2–32 months	Valproate and chemotherapy drugs	Dose, fractionation, and time
Korytko et al., 2006 [15] Retrospective *N* = 129	Both	Mixed (excluding AVMs)	SF GK SRS	12 Gy	Did not specify	23%	Did not specify	Did not specify	Every 3–6 months	N/A	Did not specify	Did not specify	Brain volume, location, previous WBRT, sex
Keller et al., 2017 [16] Retrospective *N* = 181	Metastasis	NSCLC, breast, and other mets	3 fraction GK SRS	23.1 Gy	Did not specify	18.50%	Did not specify	Did not specify	Median 15 months (range: 3–38 months	Did not specify	Did not specify	Did not specify	Location and volume
Robbins et al., 2012 [17] Retrospective *N* = 85	Metastasis	Lung (59%), Breast (11%), Melanoma/Renal (13%), Gynecologic (6%), Colon (4%), and Other (7%)	SRS to the surgical cavity (with WBRT as salvage)	12–18 Gy (median 16 Gy)	Surgery, Salvage SRS, Salvage WBRT (used in 35% of cases)	8% (7 patients)	Did not specify	Did not specify	Median 11.2 months (range: 1–93 months)	Median: 18 days (95% received SRS <2 months post-surgery)	Median: 8.4 months (range: 5.8–16.5 months)	Steroids, surgical excision, CT perfusion studies, MRI monitoring	Tumor location, radiation dose, target volume, extent of surgical resection, active systemic disease
Blonigen et al., 2010 [8] Retrospective *N* = 63 patients, 173 lesions	Metastasis	Most common: Breast and Lung	Linear accelerator-based SRS (Single-fraction)	Mean: 18 Gy (range: 12–22 Gy)	Steroid therapy (all symptomatic cases), pentoxifylline/vitamin E, hyperbaric oxygen (11%), surgical resection (33%)	14%	4%	10%	Median 13.7 months (range: 3.5–51 months)	Did not specify	Median: 11.5 months	Steroids, surgical resection, pentoxifylline, vitamin E, hyperbaric oxygen	Brain volume receiving V8 Gy–V18 Gy, conformality index, lesion size, tumor location
Minniti et al., 2011 [18] Retrospective *N* = 206 patients, 310 lesions	Metastasis	Lung (51%), Breast (18%), Melanoma (17%), Others (14%)	LINAC-based SRS (Single-fraction)	Mean: 18 Gy (range 15–20 Gy)	Steroid therapy (all symptomatic cases), high-dose dexamethasone (>4 months in 7.8% of patients), salvage WBRT (22.8%), salvage SRS (10.2%)	24% (total)	14%	10%	Median: 9.4 months (range: 2–42 months)	Did not specify	Median: 11 months (symptomatic), 10 months (asymptomatic)	Steroids, high-dose dexamethasone, hypofractionated radiotherapy, salvage SRS, WBRT	V10 Gy–V16 Gy, lesion volume, conformality index, tumor location, KPS, extracranial disease
Prabhu (2023) [19] Retrospective *N* = 404	Metastasis	NSCLC (47.3%), Breast (16.4%), Melanoma (12.3%), Renal cell (8.5%), GI (6.3%), Other (9.1%)	Single-fraction (SF) or multifraction (MF) preoperative SRS	SF-SRS: 14–17 Gy (median 15 Gy); MF-SRS: 24–30 Gy (median 24 Gy)	Surgical resection	6.7% (SF-SRS), 10.7% (MF-SRS)	Not reported	58% (SF-SRS), 75% (MF-SRS)	Steroids, surgical resection, and palliative care	SF-SRS: Median 1 day; MF-SRS: Median 2 days	Not reported	Steroids, surgical excision, and advanced imaging for differentiation	Radiation dose, lesion size, fractionation, tumor location
Imber et al., 2017 [20] Retrospective *N* = 174	Primary Brain Tumor	Glioblastoma	Gamma Knife SRS	Median: 16 Gy (range: 10–22 Gy)	Salvage craniotomy in 26.4% of patients, systemic chemotherapy, subset received bevacizumab	Salvage craniotomy (26.4%), systemic chemotherapy, subset received bevacizumab	Not reported	Not reported	Median 8.7 months (range: 0–120.1 months)	Did not specify	Median: 6.6 months (range: 1.1–83.6 months)	Steroids, surgical resection, and palliative care	Age, marginal prescription dose, treatment volume, surgery-to-Gamma Knife interval

Abbreviations: RN = radiation necrosis; SRS = stereotactic radiosurgery; HFSRT = hypofractionated stereotactic radiotherapy; WBRT = whole-brain radiation therapy; EBRT = external beam radiation therapy; LINAC = linear accelerator; GK = Gamma Knife; NSCLC = non-small cell lung cancer; Gy = gray; BED = biologically effective dose; PTV = planning target volume; HR = hazard ratio; CI = confidence interval; KPS = Karnofsky performance status; OS = overall survival; LR = local recurrence; IQR = interquartile range; MRI = magnetic resonance imaging; CT = computed tomography; AVM = arteriovenous malformation; MF-SRS = multifraction stereotactic radiosurgery; SF-SRS = single-fraction stereotactic radiosurgery; VEGF = vascular endothelial growth factor. N/A = not applicable or not reported.

**Table 2:** Radiation modalities and reported rates of impacts.

Modality	RN Rate (Glioma)	RN Rate (Metastasis)	Re-Irradiation Risk Impact	Evidence Level
WBRT	4.9% (EBRT data)	10–30%	High	RCTs and retrospective data
SRS (Single-Fraction)	15–25%	23%	>24% symptomatic RN in repeat SRS	Multiple retrospective cohorts
HFSRT (Multi-Fraction SRS)	~7%	7%	Moderate	Multicenter retrospective and ongoing RCT (NCT04114981)
IMRT	Varies, typically moderate	Variable; depends on tumor burden	Moderate	Retrospective series
Brachytherapy (I-125)	Up to 23%	17.50%	High	Early trials and retrospective data
Brachytherapy (Cs-131)	1.3–5%	1.3–4.8%	Lower with Cs-131 due to shorter half-life	Prospective single-center studies
IORT (LEX-IORT)	~2.9%	2.90%	Unknown	Phase II prospective (INTRAMET)
Proton Therapy	Lower than photons	Lower; especially in pediatric and eloquent areas	Potentially lower with Bragg peak	Phase II and registry data
Carbon Ion Therapy	Unknown due to limited data	Theoretical advantage	Potentially lower (but unclear)	Experimental, limited clinical access

WBRT: Whole Brain Radiation Therapy. SRS: Stereotactic Radiosurgery. HFSRT: Hypofractionated stereotactic radiosurgery. IMRT: Intensity Modulated Radiation Therapy. LEX-IORT: Low-Energy X-ray Intraoperative Radiation Therapy.

**Table 3:** Summary of therapeutic options for radiation necrosis following radiation therapy for central nervous system (CNS) neoplasms. HBOT, hyperbaric oxygen therapy; LITT, laser interstitial thermal therapy; PFS, progression-free survival; VEGF, vascular endothelial growth factor.

Therapy	Mechanism	Expected Duration/Response	Limitations
Corticosteroids	Reduces vasogenic edema and stabilizes vascular permeability1	Temporary benefit; median PFS ≈ 2.9 months; ~50% recurrence after discontinuation	Long term toxicity, rebound edema
Bevacizumab	Anti-VEGF antibody decreasing vascular permeability	Rapid edema reduction; 65.5% response vs 31.5% with steroids; relief in ≈ 90% on repeat cycles; some recurrence (~34%)	Hypertension; transient effect; risk of ischemia with prolonged use
Surgical resection	Physically removes necrotic tissue, relieves mass effect	Clinical improvement in > 50%; edema resolution within 2–4 weeks	Invasive; surgical morbidity; unsuitable for deep lesions
LITT	Minimally invasive laser ablation of necrotic tissue	Neurologic improvement in 50–70%; functional improvement in 70–80%	Limited data; thermalinjury risk
HBOT	Increases oxygenation and angiogenesis	70–80% improvement in small series	Potential to stimulate tumor growth; access/cost
Pentoxifylline + Vitamin E	Anti-inflammatory and antioxidant effects reduce fibrosis/necrosis	Reported radiologic improvements; adjunctive use	Limited evidence; mild side effects

## Data Availability

Not applicable.
